# Design of an internet-based health economic evaluation of a preventive group-intervention for children of parents with mental illness or substance use disorders

**DOI:** 10.1186/1471-2458-10-470

**Published:** 2010-08-10

**Authors:** Marla Woolderink, Filip Smit, Rianne van der Zanden, Jennifer Beecham, Martin Knapp, Aggie Paulus, Silvia Evers

**Affiliations:** 1 Department of Health Organization, Policy and Economics, Faculty of Health, Medicine and Life Sciences, CAPHRI, Maastricht University, P.O. Box 616, 6200 MD Maastricht, the Netherlands; 2 Centre of Prevention and Brief Intervention, Trimbos Instituut (Netherlands Institute of Mental Health and Addiction), Utrecht, the Netherlands; 3 Department of Epidemiology and Biostatistics, EMGO Institute for Health and Health Care Research, VU University Medical Centre, Amsterdam, the Netherlands; 4 Personal Social Services Research Unit, London School of Economics and Political Sciences, London, UK

## Abstract

**Background:**

Preventive interventions are developed for children of parents with mental and substance use disorders (COPMI), because these children have a higher risk of developing a psychological or behavioral disorder in the future. Mental health and substance use disorders contribute significantly to the global burden of disease. Although the exact number of parents with a mental illness is unclear, the subject of mentally ill parents is gaining attention. Moreover there is a lack of interventions for COPMI-children, as well of (cost-) effectiveness studies evaluating COPMI interventions. Innovative interventions such as e-health provide a new field for exploration. There is no knowledge about the opportunities for using the internet to prevent problems in children at risk. In the current study we will focus on the (cost-) effectiveness of an online health prevention program for COPMI-children.

**Methods/Design:**

We designed a randomized controlled trial to examine the (cost-) effectiveness of the Kopstoring intervention. Kopstoring is an online intervention for COPMI-children to strengthen their coping skills and prevent behavioral and psychological problems. We will compare the Kopstoring intervention with (waiting list) care as usual. This trial will be conducted entirely over the internet. An economic evaluation, from a societal perspective will be conducted, to examine the trial's cost-effectiveness. Power calculations show that 214 participants are needed, aged 16-25. Possible participants will be recruited via media announcements and banners on the internet. After screening and completing informed consent procedures, participants will be randomized. The main outcome is internalizing and externalizing symptoms as measured by the Youth Self Report. For the economic evaluation, healthcare costs and costs outside the healthcare sector will be measured at the same time as the clinical measures, at baseline, 3, 6 and 9 months. An extended measure for the intervention group will be provided at 12 months, to examine the long-term effects. In addition, a process evaluation will be conducted.

**Discussion:**

Recent developments, such as international conferences and policy discussions, show the pressing need to study the (cost-) effectiveness of interventions for vulnerable groups of children. This study will shed light on the (cost-) effectiveness of an online preventive intervention.

**Trial registration:**

NTR1982

## Background

This article describes the design and methods of a randomized controlled trial (RCT) evaluating the (cost-) effectiveness of an online digital preventive intervention for children of parents with a mental disorder or a substance use disorder. In Dutch this group is known as KOPP-children (Kinderen van Ouders met Psychiatrische Problemen). In English these children are often referred to as COPMI (Children of Parents with Mental Illness). Even in their adulthood, COPMI-children are regarded as a vulnerable.

The prevalence of mental illness is increasing, worldwide [1]. Accordingly, some of the newly diagnosed patients are parents. Although international statistics about global prevalence are not yet available, the subject of mentally ill parents is gaining attention [1]. In the Netherlands every year about 864,000 parents meet the criteria for a DSM III (Axis one) mental illness diagnosis [2]. In 2006 about 1.6 million COPMI-children, younger than 22 years, were living in the Netherlands [3], among a total Dutch population of 16 million. More than 700,000 of these children are adolescents between 16 and 25 years old, living with at least one parent who suffers from a mental health or substance use disorder. Internationally, the prevalence and incidence of COPMI is still unknown. The problems encountered by COPMI-children have a large impact on their lives, their social environment and on society as a whole. COPMI-children have, overall, a lower quality of life, and are likely to use health care and social care facilities frequently. In addition participation in school and work can be a problem [4]. Previous studies in this field showed that COPMI-children have 50% likelihood of developing mental health problems themselves throughout life [5]. If a child has two (biological) parents with mental health or substance use disorders, the likelihood rises to 66% [3]. During their adolescence, two out of three COPMI-children experience serious problems in coping with a parental manifestation of mental or substance use disorder [4]. There is a growing interest in COPMI children, both nationally, as well as internationally. Recently, an international conference about the forgotten child, stressed the importance of children of parents with mental illness [1]. COPMI have also come to the attention of politicians. In May 2009, for example, members of the Dutch parliament asked formal questions in the House of Representatives about the increasing prevalence of COPMI-children, the consequences of their situation and what solutions were needed. Thus, societal impact and political interest is growing nationally and internationally.

To date, only two studies have examined the effectiveness of COPMI interventions. As a consequence most of the interventions developed for this group have not yet been evaluated and there is no evidence relating their cost-effectiveness [6].

Two COPMI interventions used on a large scale in the Netherlands and elsewhere are based on face-to face psycho-educative family intervention. Literature shows that these psycho-educative interventions are effective in terms of protective factors [7,8].

In spite the elevated risks, only a few prevention programs are offered for COPMI-children and these reach only part of the target population. One of the options for improving accessibility is providing internet-based interventions (E-programs). In the Netherlands an E-program called Kopstoring has recently been developed.

In brief the Kopstoring intervention is an online 8-week educative course which strengthens the coping skills of COPMI-children, and provides knowledge about their parents' mental illness. In a secure chat box COPMI-children work through the themes of the course. Kopstoring is an online intervention for COPMI children aged 16 to 25 years.

In this study we will examine the (cost-) effectiveness of the Kopstoring E-program for COPMI-children. To our knowledge this is the first study to assess the (cost-) effectiveness of E-programs for COPMI-children. Besides the information regarding cost-effectiveness, this study will also yield information about COPMI-children's use of healthcare resources.

This study builds on an existing COPMI pilot study which assesses the effects of the online intervention [9]. E-mental health is a relatively new method for delivering interventions in the health care sector at affordable costs. A series of recent studies [10] have demonstrated the positive effects of E-mental health interventions and highlighted features such as therapists being able to delegate routine aspects of their work to computers, thus freeing scarce resources for those patients most in need of it, and delivering interventions in a well structured way based on the best available evidence [11]. Several systematic reviews [12,13] have identified the effectiveness of E-mental health interventions. Despite a promising trend several authors stresses that both the effectiveness as well as the costs-effectiveness of E-mental health interventions need to be assessed [12,14].

In brief, the current study aims to examine the beneficial effects of online intervention for COPMI-children, weighing these against health and societal costs. Potentially, a preventive Kopstoring program will reduce symptoms, behavioural and serious psychological problems, strengthen the COPMI-children's emotional and social functioning and coping skills, and improve their relationship with the ill parent. A decrease in the consumption of health care may follow. Production losses, leaving school prematurely, vandalism and costs related to service use outside the health care sector (social care) are also expected to be lower. This study will lead to more solid evidence-based information about the Kopstoring program. This study will be the first to provide insight into the cost-effectiveness of a preventive E-program for COPMI-children.

## Methods/Design

The design will be a multi-centered RCT. The project will consist of an effectiveness study, an economic evaluation, and a process evaluation.

### Objective and research questions

The main study objective is to evaluate the effects, cost-effectiveness and the process of providing an internet-based preventive intervention for COPMI-children from 16-25 years old.

The main research question is as follows: From a societal perspective, is the online psycho-educative prevention program Kopstoring a cost-effective program for reducing the symptoms of children of parents with mental or substance use disorders? In addition, is the intervention delivered in time and according to prevailing standards, according to the protocol and does it meet the expectations of caregivers and participants involved?

The following sub-questions can be indentified for the effectiveness study, the economic evaluation study and process evaluation:

### Effectiveness study

1. Does the preventive online intervention reduce the child's internalizing and externalizing symptoms and prevent behavioural and coping problems which are due to the parent's mental illness?

2. Does the preventive online intervention strengthen the child's emotional and social functioning and coping skills?

3. Does the preventive online intervention establish a higher quality of life for COPMI-children?

### Economic evaluation study

1. From a societal perspective, is the delivery of the Kopstoring intervention, in comparison with waiting-list care as usual, preferable in terms of costs, effects and utilities?

### Process evaluation

1. Is the intervention delivered in time and does it meet accepted standards, according protocol?

2. Is the protocol being followed?

3. What do participants expect from the intervention and what do they believe about the effect of the intervention (credibility and expectancy)?

### Design

This study will consist of an effectiveness study, an economic evaluation study and a process evaluation.

**Figure 1:**
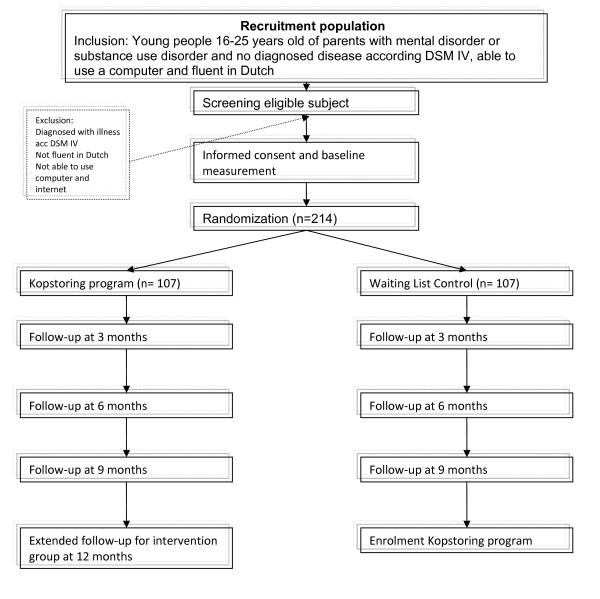
**Flow chart study design**.

The basis of the effectiveness study will be a pragmatic randomized controlled trial (RCT), in which the Kopstoring program will be compared with a waiting list control condition which reflects care as usual (CAU). The interventions last 8 consecutive weeks with a ninth meeting after a month. Figure 1 presents the flow chart for the study. Cost and outcome measures will be taken at baseline and at 3 months and 6 months after baseline in both conditions. An extended follow-up at 12 months will be conducted in the intervention arm of the trial, to monitor the long-term costs and effects of the intervention.

In the economic evaluation we will compare incremental costs and incremental outcomes of the Kopstoring intervention in relation to care as usual. This economic evaluation will involve both a cost-effectiveness analysis (CEA) and a cost-utility analysis (CUA). The primary outcome measure for the economic evaluation will be the prevention of the development of symptoms over the 6 months of follow-up. Within the cost-utility analysis, outcomes will be measured by means of the standard Dutch version of the EuroQol. In the CEA, the incremental cost-effectiveness ratio (ICER) will be expressed as the incremental costs per point improvement on the Youth Self Report Questionnaire. The primary outcomes measured for the cost-utility analysis will be QALYs, based on the EuroQol utility scores [15,16]. In the CUA, the ICER will be expressed as the incremental cost per QALY. This economic evaluation will take a societal perspective, including all relevant costs and outcomes.

### Process evaluation

During the study we will include a process evaluation in which we assess several aspects concerning the process of the execution of the intervention and how it is perceived by caregivers and the recipients. We want to identify whether the execution of the RCT is going according to protocol and if so, whether the intervention is meeting accepted standards [17]. We will use qualitative methods to derive this information. We will interview the experts (caregivers) who are involved in the delivery of the Kopstoring program to gain knowledge about the factors which harm or stimulate working according to protocol. The recipients will receive a short questionnaire with items about aspects of the intervention.

Credibility and expectations about internet interventions and specifically the Kopstoring intervention will be measured at baseline and after receiving the internet intervention. These results will be used for the process evaluation to gain knowledge about what respondents expect from this internet therapy and how they rate it in terms of credits. Caregivers will also be asked to fill in the Credibility and Expectancy Questionnaire. In addition the statistics linked to the database system can be useful in determining whether the protocol was followed

### Target population

The target group is defined as young people aged between 16 and 25 years who have at least one parent suffering from a psychiatric disorder, multiple psychological disorders or an addiction problem. For inclusion in our study, young people have no internalizing and externalising problems. This will be defined using the Youth Self report List (YSR); the cutoff scores for girls will be a score of 21 and for boys a score of 16. If the young person has a score above the cutoff score, they will be referred for treatment or other services. In addition, the young person has to be sufficiently fluent in the Dutch language, have access to the internet and be able to use a chat box - to participate actively and be able to listen to other participants. For young people aged 16-17 informed consent from the parent is also required. For those aged 18 and over, informed consent of the adolescent is sufficient. Those who are younger than 16 years or older than 25 years are excluded from our study.

### Recruitment

COPMI-children will be recruited in several ways. First, they can be recruited through, the online Kopstoring program (http://www.kopstoring.nl). This open website has information about the study and its objectives. Second, the mental health institutions who are cooperating in this project and provide the online program give information about the Kopstoring program to potential respondents and can recruit young people through their websites, databases, networks and contacts. General practitioners (GPs) will be informed about the study so they can also preselect eligible respondents. There will also be banners and links created for the internet. Public Relations (PR) will be led by the Trimbos Institute which has experts and experience in the field of recruitment and PR.

### Intervention

The Kopstoring program consists of 8 consecutive online weekly chat group meetings and a ninth evaluation meeting in a chat box at the website http://www.kopstoring.nl. The aim of the intervention is to strengthen protective factors, such as self-management skills and psychological well-being, and prevent the development of psychological disorders. The experts (prevention workers and psychologists from mental health care institutions) involved in the delivery of the intervention are specialized in the field of COPMI-problems and in the prevention of mental health problems in young people. The intervention is protocol-driven and well-structured. There are 8 themes, so each week there is a new theme. The themes are; getting acquainted with the home situation, roles in families, thoughts and feelings, questions about addiction and mental problems, different styles of behaviour, social networks, leading your own life in relation to social networks and what is coming up in the future. The participants are required to carry out homework assignments before entering the chat box (chat room) meeting [18]. The Kopstoring program is part of an informative website which also provides an e-mail service and chat options, alongside the Kopstoring program.

### Care as usual in the Netherlands

The control condition in this trial is a waiting list with unrestricted access to care as usual. Usual care in the Netherlands is a mix of several services. In this study, care as usual is not standardized and there are no protocols. Young people can request a lot of services: for example, a general practitioner, a psychologist, or mental health or institutional services such as counselling sessions.

### Sample Size calculation

The sample size calculation is based on the ability to detect a medium size or larger clinical effect. This corresponds to a standardized mean difference (Cohen's d) of d > 0.45 [19]. We will test the hypotheses at a conventional power of (1 - beta) = 0.80 and alpha = 0.05, 2-tailed (cf. Friedman et al., 1996). For this n = 78 is required per condition, therefore a total of 156 participants. Finally we want to compensate for possible drop-out during the trial. The drop-out rate from the pilot study was 28% [9]. We therefore need to enrol 156/(1-0.28) = 214 participants during the trial.

### Cost and outcome measurement

#### I) Effectiveness study

Clinical outcomes will be measured using various questionnaires. The primary outcome of the clinical effect study is the symptoms, complaints and well-being of the young person measured with three questionnaires, each focussing on a different aspect of symptoms, complaints and well-being.

### Youth Self Report List (YSR) of the Children Behaviour Checklist

The Youth Self Report List will be used to measure each subject's competencies and behavioural problems. The instrument is proven to be valid and has a good internal consistency [20,21]. The Youth Self Report List (YSR) is part of the Child Behaviour Checklist which is a questionnaire measuring internalizing and externalizing psychological problems in young people [20,21]. The Self-Report List has three versions: one each for parents, teachers and young people. For this study we use the young person's version.

### Secondary clinical outcome

The Effect Evaluation List (EEVL) is a Dutch questionnaire tailor-made for the online Kopstoring program: it measures several aspects of coping skills and functioning. The instrument consists of 8 items in two main dimensions: biographical information about the respondent (19 items) and the effect of the COPMI intervention (61 Items). The EEVL has been proven to be valid and has good reliability [22].

### Quality of Life (EuroQol 5D)

The EQ-5D is a self-administered questionnaire. Both generic quality of life, as well as utilities, will be derived by means of the EQ-5D, which will be completed by the young people. The EQ-5D has been chosen because it is a widely used quality of life instrument (nationally and internationally) and its reliability and validity is well established [23]. The EQ-5D contains 5 dimensions of health-related quality of life: mobility, self-care, daily activities, pain/discomfort and depression/anxiety. Each dimension can be rated on three levels: no problems, some problems and major problems. In addition, the EQ-5D consists of a visual analogue scale (VAS) ranging from zero (worst imaginable health state) to 100 (best imaginable health state).

### Utilities

Utilities will be derived from the EQ-5D using the following steps. The 5 dimensions will be summarized into a health state. Utility values will be calculated for these health states, using preferences elicited from a general population, the so-called algorithms. One algorithm has been established using a general population from the UK, the Dolan tariff [24]. For the sensitivity analysis the Dutch algorithm will be used [25]. The utility values derived will be used to compute Quality Adjusted Life Years (QALYs). A QALY combines survival and utilities. The utilities at the three time points will be used to compute the Quality Adjusted Life Years gained (QALY) using of the area under the curve method.

### Symptom Checklist (SCL-90)

The Symptom Checklist has 90 items, each answered using 5 options covering 8 dimensions. These dimensions are: phobia, anxiety, depression, physical complaints, insufficient acting and thinking, distrust and interpersonal sensitivity, hostility and sleeping problems. The SCL-90 is a psychopathology-indicator and covers both somatic and psychological symptoms. It takes about 20 minutes to complete and has been tested and appears to be a valid instrument [26].

#### II) Economic evaluation study

In this economic evaluation we collect data on service use to derive the costs of using these services.

Collecting data on service use:

##### Resource use questionnaire

The use of the Kopstoring program will be measured as well as the use of other resources both within and outside the healthcare sector. A questionnaire will be developed to measure the use of resources.

##### Productivity losses (PRODISQ)

Production losses will be measured using the PROductivity losses and DISability Questionnaire (PRODISQ), a module-based questionnaire. This questionnaire will be used only for those with a paid job. Three modules will be used for this study to gather data on absenteeism and presenteeism. Production costs will be calculated by using the friction cost method [27,28].

##### Productivity losses (PRODISQ) and School absenteeism

For the young people who go to school, we adjusted the same three modules from the PRODISQ as we did to measure absentieesm and presenteeism among the working young persons.

#### III) Process evaluation

##### Qualitative Interviews

Interviews will be conducted with the caregivers of the mental health services who provide the online Kopstoring program. We will ask them questions about offering the program as described in the protocol and about the process of enrolment. In addition to conducting these qualitative interviews with caregivers, we will send a short online questionnaire to the program respondents.

##### Credibility/Expectations Questionnaire (CEQ)

The credibility and expectations questionnaire, a 6-item list, is used to determine what the respondents expect from the online program and how many credits they give the intervention.

Credibility and Expectations will be measured by the Credibility/Expectations Questionnaire (CEQ) which consists of 6 items using a 9- or sometimes 10-point Likert scale. This instrument is meant to measure the expectations and credibility a person has about the received intervention [29]. The questionnaire can be used at baseline and during follow-up to compute the different scores in expectation and credits one gives to a particular therapy. In their article Borkovec et al show that the instrument has proven to be valid and that the psychometric properties are of good quality[30]. It will take around five minutes to complete the 6 items of this instrument.

### Analyses

Data will be analysed according to the intention-to-treat principle, which means including data from all participants irregardless of whether they received the intervention or not. Missing data on the item level will be handled using SPSS missing values analysis. Completely missing measurements will be handled using multiple imputation. A baseline analysis will be performed to examine the comparability of groups at baseline for both costs and outcomes. Differences in baseline will be controlled using applied methods to correct these differences [31]. Losses to follow-up will be described.

The analysis consists of comparing the assessments and scores of the respondents on the questionnaires. Clinical outcomes after the intervention (8 weeks), at three months, six months and twelve months will be compared between groups and within groups, which means we want to compare the effect of the intervention in respondents in the intervention and control groups and assess the additional effects before and after receiving the intervention.

#### II) Economic evaluation study

The economic analysis will also be performed according to the intention-to-treat principle. Handling of missing data will be similar to the effectiveness study. A baseline analysis for the economic evaluation will also be performed to examine the comparability of groups at baseline for both costs and outcomes. If necessary methods will be applied to control for differences in baseline [31]. Despite the usual skewness in the distribution of costs, the arithmetic means are generally considered to be the most appropriate measures to describe cost data [32]. Therefore arithmetic means (and standard deviations) will be presented. Non-parametric bootstrapping will be used to test for statistical differences in costs between the intervention and control group. Non-parametric bootstrapping is a method based on random sampling with replacement based on the participants' individual data [33]. The bootstrap replications will be used to calculate 95% confidence intervals around the costs (95% CI), based on the 2.5th and 97.5th percentiles.

The incremental cost-effectiveness ratio (ICER) will be determined on the basis of incremental costs and the effects of the Kopstoring program in comparison with care as usual. The cost-effectiveness ratio will be stated in terms of costs per unit of outcome; the cost-utility ratio will focus on the incremental cost per QALY gained. The ICER will be calculated as follows. ICER = (Ci - Cc)/(Ei - Ec), where Ci is the annual total cost of the Kopstoring group, Cc is the annual total cost of the care as usual group, Ei is the effects at the 6-month follow-up for the Kopstoring group and Ec is the effect at the 6-month follow-up for the care as usual group. The robustness of the ICER will be checked by non-parametric bootstrapping. Bootstrap simulations will also be conducted in order to quantify the uncertainty around the ICER, yielding information about the joint distribution of cost and effect differences. The bootstrapped cost-effectiveness ratios will be plotted subsequently in a cost effectiveness plane, in which the vertical line reflects the difference in costs and the horizontal line reflects the difference in effectiveness. The choice of treatment depends on the maximum amount of money that society is prepared to pay for a gain in effectiveness. Therefore, the bootstrapped ICERs will also be depicted in a cost-effectiveness acceptability curve, showing the probability that Kopstoring is cost-effective using a range of ceiling ratios. To demonstrate the robustness of our base-case findings a multi-way sensitivity analyses will be performed in which assumptions in the base case analysis will be recalculated to assess whether they have influenced the incremental cost-effectiveness ratio (ICER), for example by varying unit costs and volumes between minimum and maximum [33]. The bootstrap replications will be used to calculate 95% confidence intervals around the median costs, while the 95% CI will be based on the 2.5th and 97.5th percentiles.

#### III) Process evaluation

We will monitor whether certain aspects are going according to protocol and on schedule by qualitative interviews, data assessment and the use of the Credibility and Expectancy questionnaire.

The analysis will consist of the following steps:

1) Analyzing the qualitative interviews

2) Comparing the results of the analysis with the standards and requirements in the protocol

3) Identifying factors where the delivery of the intervention deviates from protocol (factors that harm working according protocol) and factors that stimulate working according to protocol (success factors)

4) Analyzing the participants' expectations and beliefs in the effects of the intervention according the C&E questionnaire.

### Collaboration

The initiative for this study came from Maastricht University working together with Trimbos Institute. This study will be conducted with support from professionals from several disciplines. The London School of Economics will be involved in the academic part of the study. Mental health institutions in the Netherlands will be involved in the delivery of the online preventative intervention, including Dimence (Deventer), GGNet (Warnsveld), De Gelderse Roos (Wolfheze), GGNet Apeldoorn, Dimence Almelo, GGZ Oost Brabant, De Grote Rivieren and GGZ Zuid Holland. The Trimbos Institute will be responsible for developing a PR-strategy and will be involved with the recruitment of participants.

This study is registered in the Netherlands Trial Register, part of the Dutch Cochrane Centre (NTR1982). The ethical committee from Maastricht University Academic Hospital has given ethical approval for performing the experiment.

## Discussion

The field of E-mental health and internet therapies is growing. Many new therapies have been developed, but few have been evaluated. A lot of them are preventative, so there will be no direct harm if effectiveness is not established. The implementation of evidence-based medicine means that the effectiveness and costs of new therapies and interventions should be established. This study will provide information about E-interventions in Dutch society in general, but will also yield specific information about a vulnerable group of young people in society. In the event that we can show effectiveness and cost-effectiveness, the aim will be to implement this intervention as standard care in the Netherlands.

### Methodological considerations

Interventions in the field of COPMI-problems are relatively new and internet interventions have scarcely been evaluated. Internet-driven research has the advantage of being anonymous, but it can be hard to keep participants in the study because there is no face-to face contact with the participants. The participants in this study are 16-25 years old. When a minor is involved in scientific research, the informed consent of the parents as well as of the minor is needed according to Dutch Law (WMO art. 4). This requirement for extended consent might lead to the under representation of younger participants, as they might not want to inform their parents about participating in the study and therefore be unable to get their consent.

### Feasibility

Feasibility was a very important key point in writing the proposal for the study. To establish effectiveness 214 respondents are needed. To ensure we could recruit sufficient young people we devised a PR-strategy including high quality advertisements. Links banners and other methods for reaching the target population, will be used. A special budget has been reserved for this purpose.

## Conclusion

Recent developments show the pressing need for studying the effectiveness of interventions for children of parents with a mental illness or substance use disorder. In a meeting of the House of Representatives in May 2009, critical questions were asked about the growing prevalence of COPMI-children and the lack of good interventions [34]. While E-mental health interventions are likely to be easily accessed by young people, there is a pressing need to study the (cost)-effectiveness of these recent developments in health care. There is hardly any evidence, but what exists is promising [12]. This study builds on this evidence and provides a unique opportunity for developing research techniques in this new area, as well as supporting the provision of better services for COPMI.

## Competing interests

The authors declare that they have no competing interests.

## Authors' contributions

All authors participated in describing the design of this study. MW, SMAAE, FS, RvdZ obtained funding for this study. MK, JB and AP corrected the proposal and the manuscript extensively. All authors drafted this manuscript. All authors have read and approved this manuscript.

## Pre-publication history

The pre-publication history for this paper can be accessed here:

http://www.biomedcentral.com/1471-2458/10/470/prepub

## References

[B1] The forgotten child, EUFAMI http://eufami.org

[B2] Association APA Diagnostic and statistical manual of mental disorders, (DSM) 1994

[B3] Factsheet Preventie 2: Kinderen van ouders met psychische problemen http://www.trimbos.nl

[B4] Factsheet Online Groepcursus Kopstoring http://www.trimbos.nl

[B5] Rutter M Quinton D Parental psychiatric disorders: effects on children Psychological Medicine 1984 14 853 880 10.1017/S0033291700019838 6545419

[B6] Fraser CE Anderson K Lloyd D Judd F Intervention programs for children of parents with a mental illness: a critical review International Journal of health Promotion 2006 8 8 19

[B7] Beardslee W Gladstone T Wright E Cooper A A Family-Based Approach to the Prevention of Depressive Symptoms in Children at Risk: Evidence of Parental and Child Change Pediatrics 2003 112 e119 e131 10.1542/peds.112.2.e119 12897317

[B8] Beardslee W Ellen M Wright E Salt P Drezner K Gladstone T Versage E Rothberg P Examination of children's response to two preventive intervention strategies over time Journal of the American Academy of Child and Adolescent psychiatry 1997 36 196 204 10.1097/00004583-199702000-00010 9031572

[B9] Veen C Van Der Zanden R Procesevaluatie Kopstoring 2007 Utrecht: Trimbos Insituut

[B10] Beardslee WR Keller MB Lavori PW Klerman GK Dorer DJ Samuelson H Psychiatric disorder in adolescent offspring of parents with affective disorder in a non-referred sample J Affect Disord 1988 15 3 313 322 10.1016/0165-0327(88)90028-6 2975303

[B11] Fact sheet I.COM http://www.trimbos.nl

[B12] Kathenthaler E Schackley P Stevens K Beverly C Parry G Chilcott J A systematic review and economic evaluation of computerized cognitive behaviour therapy for depression and anxiety Health Technology Assessment 2002 6 22 10.3310/hta6220 12433315

[B13] Marks IM Cavanagh K Gega L Computer-aided Psychotherapy: Revolution or bubble? BRITISH JOURNAL OF PSYCHIATRY 2001 191 471 473 10.1192/bjp.bp.107.041152 18055948

[B14] Marks IM Cavanagh K Gega L Hands-on help: computer-aided psychotherapy 2007 New York: Psychology press

[B15] Brooks R EuroQol Group Euroqol: the current state of play Health Policy 1996 37 53 72 10.1016/0168-8510(96)00822-6 10158943

[B16] The EuroQol Group EuroQol - a new facility for the measurement of health-related quality of life Health Policy 1990 16 3 199 208 10.1016/0168-8510(90)90421-9 10109801

[B17] WHO Workbook 4 Process Evaluations http://www.unodc.org/docs/treatment//process_evaluation.pdf

[B18] Leunissen M Rosenbrand R Zanden van der AP Online cursus Kopstoring Een preventief groepsaanbod via internet voor jongeren (16-25 jaar) van ouders met psychische en/of verslavingsproblematiek 2007 Utrecht: Trimbos Institute

[B19] Lipsey MW Wilson DB The efficacy of psychological, educational, and behavioral treatment American Psychologist 1993 48 1181 1201 10.1037/0003-066X.48.12.1181 8297057

[B20] Achenbach TM Manual for the Child Behavior Checklist/4-18 and 1991 Profile 1991 Burlington VUoV, Department of Psychiatry

[B21] Achenbach TM Integrative Guide to the 1991 CBCL/4-18, YSR, and TRF Profiles 1991 Burlington VUoV, Department of Psychology

[B22] Huijnen SEA Valkenberg IMC Handleiding bij de Effect Evaluatie Lijst voor KOPP/KVO-preventiegroepen voor adolescenten 2005 Utrecht: Trimbos

[B23] Brooks R The EuroQol: The current state of play Health Policy 1996 37 1 53 72 10.1016/0168-8510(96)00822-6 10158943

[B24] Dolan P Modeling valuations for EuroQol health states Medical Care 1997 35 1095 1108 10.1097/00005650-199711000-00002 9366889

[B25] Lamers LM Stalmeier PFM McDonnell J Krabbe PFM Van Busschbach JJ Kwaliteit van leven meten in economische evaluaties: het Nederlands EQ-5D-tarief Nederlands Tijdschrift voor Geneeskunde 2005 149 1574 1578 16038162

[B26] Arrindell WA Ettema JHM Handleiding bij een multidemensionele psychopathologie-indicator 1986 Lisse: Swets &&; Zeitlinge

[B27] Koopmanschap M PRODISQ: a modular questionnaire on productivity and disease for economic evaluation studies Expert review of Pharmacoeconomics and Outcome research 2005 5 23 28 10.1586/14737167.5.1.23 19807557

[B28] Koopmanschap M Meerding W Evers S Severens J Burdorf A Brouwer W Handleiding voor het gebruik van PRODISQ. Een modulaire vragenlijst over de relatie tussen ziekte en productiviteitskosten. Toepasbaar bij economische evaluaties van gezondheidszorgprogramma's voor patiënten en werknemers 2004 Rotterdam/RMEU, Maastricht EMCU

[B29] Borkovec T Nau S Credibility and of analogue therapy rationales Journal of Behaviour Therapy and Experimental Psychiatry 1972 3 257 260 10.1016/0005-7916(72)90045-6

[B30] Devilly GJ Borkovec TD Psychometric properties of the credibility/expectancy questionnaire Journal of Behavioral Therapy and Experimental Psychiatry 2000 31 73 81 10.1016/S0005-7916(00)00012-4 11132119

[B31] Manca A Hawkins N Sculpher MJ Estimating mean QALYs in trial based cost-effectiveness analysis: the importance of controlling for baseline utility Health Economist 2005 14 5 487 496 10.1002/hec.944 15497198

[B32] Ramsey S Willke R briggs AH Brown R Buxton M Chawla A Cook J Glick H Liljas B Pettini D Good reserach for cost-effectiveness analasys alongside clinical trial: The ISPOR RCT-CEA Task Force report Value in Health 2005 8 5 821 833 10.1111/j.1524-4733.2005.00045.x 16176491

[B33] Briggs AH wonderling DE Mooney CZ Pulling cost-effectiveness analysis by bootstraps: a non-parametric approach to confidence interval estimation Health Economist 1997 6 4 327 340 10.1002/(SICI)1099-1050(199707)6:4<327::AID-HEC282>3.0.CO;2-W 9285227

[B34] Ministerie van Volksgezondheid WeS Ministerie van Volksgezonheid WeS Kamervragen 18 mei 2009: Antwoorden op kamervragen van Bouwmeester en Bouchibiti over kinderen van ouders in de geestelijke gezondheidzorg (GGZ) en de forensische GGZ 2009 Den haag

